# iTRAQ proteomic analysis of the anterior insula in morphine‐induced conditioned place preference rats with high‐frequency deep brain stimulation intervention

**DOI:** 10.1111/adb.70014

**Published:** 2025-01-21

**Authors:** Haigang Chang, Yaxiao Wang, Lei Hui, Yuling Diao, Pengju Ma, Xiangsheng Li, Feng Wang

**Affiliations:** ^1^ Department of Neurosurgery The First Affiliated Hospital of Xinxiang Medical University Weihui China; ^2^ Department of Ultrasound The First Affiliated Hospital of Xinxiang Medical University Weihui China; ^3^ Department of Neurosurgery, The First Affiliated Hospital Zhejiang University School of Medicine Hangzhou China

**Keywords:** anterior insula, deep brain stimulation, iTRAQ, morphine addiction, proteomics

## Abstract

Morphine dependence or addiction is a serious global public health and social problem, and traditional treatments are very limited. Deep brain stimulation (DBS) has emerged as a new potential treatment for drug addiction. Repeated use of morphine leads to neuroadaptive and molecular changes in the addiction‐related brain regions. We have previously performed isobaric tags for relative and absolute quantitation (iTRAQ) labelling coupled with 2D‐LC MS/MS in anterior insular samples from rats treated with saline control, morphine or morphine plus DBS, and the identified expression of eight proteins are altered by morphine and reversed by high‐frequency DBS (HF‐DBS). In this study, we analysed the proteomic data in more details. A total of 5575 proteins were identified. Relative to the saline group, the morphine group showed 14 down‐regulated and three up‐regulated proteins. There were 118 proteins increased and 87 proteins decreased between DBS implanted animals and morphine group. Several differentially expressed proteins were verified with parallel reaction monitoring (PRM) assay. Based on Gene Ontology enrichment an KEGG pathway analyses, the majority of these differentially expressed proteins (DEPs) were involved in protein metabolic process, G‐protein coupled receptor signalling pathway, calcium‐mediated signalling, neurotransmitter transport, dopaminergic synapse and mTOR signalling pathway. These data offer a comprehensive understanding of the proteomic changes associated with morphine addiction and DBS therapy in addicted animal models, which is important for the development of DBS interventions for drug addiction.

## Graphical abstract

Drug addiction is a public health concern in the world, and deep brain stimulation is the most promising treatment in recent years, but its specific mechanism has not been clarified. In this paper, proteomics technology was used to discover the key change proteins and key signal pathways of deep brain stimulation in the treatment of drug addiction, which explains the molecular mechanism of deep brain stimulation, and provide key molecules and signal pathways for exploring more treatment techniques.

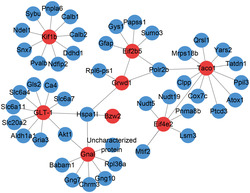

## 1 INTRODUCTION

Opioid drugs are widely used as effective analgesics for chronic, moderate‐to‐severe pain relief.^1^, ^2^, ^3^ However, chronic opioid use often leads to misuse and addiction.^4^, ^5^ Opioid addiction has rapidly become a global public health and social concern.^6^, ^7^ Morphine, a representative opioid, is commonly prescribed in clinical settings and often results in morphine dependence or addiction.^8^ Therefore, understanding the molecular and functional mechanisms of morphine addiction is crucial for developing less addictive alternatives for morphine treatment.

The concept of the ‘Morphinome’, as proposed by Bodzon‐Kulakowska et al. aims to explore the proteomic changes associated with morphine addiction and uncover its molecular mechanisms.^9^ In recent years, various research groups have identified proteins involved in morphine addiction. The Morphinome Database (www.addictionproteomics.org) was established to compile information on proteins regulated under the influence of morphine, aiding in the exploration of the molecular basis of morphine addiction.^10^ Data from 29 published articles until 2018 were included in this database. Many of these studies employed two‐dimensional electrophoresis (2DE) analysis to examine protein expression profiles. For instance, 2‐DE was used to assess the differential expression profile of prefrontal cortex (PFC) synapses in morphine‐induced conditioned place preference (CPP) rats.^11^ Different proteins were identified using the immobilized pH gradient 2‐DE in the hippocampal tissue of rats experiencing morphine addiction recurrence^12^ or during chronic morphine treatment and withdrawal.^13^ Proteomic analysis in other brain regions, including nucleus accumbens,^14^, ^15^ ventral tegmental area^16^ and amygdala,^17^ also utilized 2‐DE in different animal models or at different stages of addiction, but few studies have explored the insula's proteomics in morphine addiction animal models.

The insula, a hub of interoception, plays a crucial role in drug addiction development and maintenance, contributing to all three addiction stages.^18^, ^19^, ^20^, ^21^ Investigating insula proteomics enhances our understanding of the structural and functional changes associated with morphine addiction at the molecular level.

Current opioid addiction treatment includes μ‐opioid agonist, partial agonist and antagonist medications.^22^ However, these medications are not as widely as widely as needed. Deep brain stimulation (DBS), which modulates neuronal activity in specific brain circuits, is a reversible, adjustable, minimally invasive and safe neurosurgical intervention that has been applied successfully in treating neuropsychiatric disorders such as Parkinson's disease,^23^, ^24^ dystonia,^25^ obsessive‐compulsive disorder^26^ and severe depressive disorder.^27^ Preclinical and clinical studies suggest that DBS holds promise as an intervention for drug addiction, although its specific molecular mechanisms in drug addiction remain unclear.^28^ Therefore, proteomic analysis of the insula following DBS intervention in morphine‐addicted animals is expected to elucidate the molecular mechanisms involved in morphine addiction treatment and DBS intervention. This will contribute to a more comprehensive understanding of the biological processes underlying DBS in the treatment of neurological diseases.

In this study, we further analysed the proteomic data of saline control, morphine and morphine with DBS groups from our recently published study^29^ and identified the signalling pathways involved in morphine addiction and DBS intervention.

## 2 MATERIALS AND METHODS

### 2.1 Animals, DBS surgical implantation and morphine‐conditioned place preference (CPP)

Male Sprague–Dawley rats, weighing 260–280 g (6‐8 weeks), were housed at the standard temperature of 23°C–29°C and 12‐h light/dark cycle (7:00 AM–7:00 PM) for free access to food pills and tap water. Experimental rats for measuring morphine preference were allocated to three different groups: (1) the saline group (*n* = 15); (2) the morphine group that received alternate saline and morphine, without deep brain stimulation (DBS) apparatus implantation (*n* = 15); and (3) the morphine–DBS group that received alternate saline and morphine, with DBS apparatus implantation and continuous electrical stimulation in every experiment phase (*n* = 13). The rats were acclimated to the laboratory environment for 1 week. The behaviour experiment was carried out in the semidarkness condition. All procedures were approved by the Animal Ethics Committee of Ningxia Medical University. Morphine hydrochloride was purchased from Shenyang First Pharmaceutical Factory (10 mg/mL, Shenyang, China). Morphine‐conditioned place preference was established at a dose of 10 mg/kg (s.c) as in previous studies.^30^, ^31^ The surgical implantation of DBS apparatus and morphine‐conditioned place preference (CPP) were previously described.^29^

### 2.2 iTRAQ proteome analysis

#### 2.2.1 Protein preparation

The animals (three rats from each saline, morphine and morphine–DBS groups) were anaesthetised by inhalation of isoflurane (3%–5%) and decapitated immediately after the last behavioural test, and the whole brain was removed. The meninges and residual blood were removed in ice physiological saline and then put into the brain slice mould of rats. One‐millimetre‐thick slices close to the electrode channel were obtained. According to the brain atlas of rats (reference), the left and right anterior insula were cut and placed in 1.5 mL EP tube, weighed and recorded, immediately put into the liquid nitrogen tank for 5 min, and quickly transferred to −80°C frozen storage.

#### 2.2.2 Protein extraction

The tissue samples were taken from the refrigerator at −80°C, ground into powder at low temperature, transferred to the centrifugal tube precooled with liquid nitrogen, lysed with a proper amount of protein cracking liquid (100 mM ammonium bicarbonate, 8 M urea, 0.2% SDS, pH = 8), shaken and mixed evenly and full cracked by ultrasound in an ice water bath. The lysate was centrifuged at 4°C and 12 000 g for 15 min, and the supernatant was added with 10mM DTT at 56°C for 1 h, followed by a sufficient amount of IAM at room temperature for 1 h in the dark. The samples were completely mixed with four times the volume of precooled acetone by vortexing and incubated at −20°C for at least 2 h, then centrifuged and the precipitation was collected. After washing twice with cold acetone, the pellet was dissolved by dissolution buffer which contained 0.1 M triethylammonium bicarbonate (TEAB, pH 8.5) and 6 M urea.

#### 2.2.3 Protein quantification

The protein precipitate was dissolved with a proper amount of protein solution (6 M urea, 100 mM TEAB, pH = 8.5), and the protein concentration was determined by Bradford protein quantitative kit.

#### 2.2.4 iTRAQ labelling of peptides

One hundred and twenty micrograms of each protein sample was taken, and the volume was made up to 100 μL with dissolution buffer. Then, 1.5 μg trypsin and 500 μL of 100 mM TEAB buffer were added, and the sample was mixed and digested at 37°C for 4 h. A total of 1.5 μg trypsin and CaCl_2_ were added, and the sample was digested overnight. Formic acid was mixed with digested sample, adjusted pH under 3, and centrifuged at 12 000 g for 5 min at room temperature. The supernatant was slowly loaded to the C18 desalting column, washed with washing buffer (0.1% formic acid, 3% acetonitrile) three times and then eluted by some elution buffer (0.1% formic acid, 70% acetonitrile). The eluents of each sample were collected and lyophilized. Twenty microlitres of 1 M TEAB buffer was added to reconstitute, and enough iTRAQ labelling reagent (dissolved in isopropanol) was added; the sample was mixed with shaking for 2 h at room temperature. Then, the reaction was stopped by adding 100 μL of 50mM Tris–HCl (pH = 8). All labelling samples were mixed with equal volume, desalted and lyophilized.

Separation of fractions of peptide mixtures labelled with iTRAQ was performed using mobile phases A (2% acetonitrile, adjusted pH to 10.0 using ammonium hydroxide) and B (98% acetonitrile) for gradient elution. The peptide mixture was dissolved in mobile phase A (2% acetonitrile, 98% water, adjusted to pH = 10) and centrifuged at 4°C for 20 min at 14 000 g. The sample was fractionated using a C18 column (Waters BEH C18 4.6 × 250 mm, 5 μm) on a Rigol L3000 HPLC system; the column oven was set to 50°C. Finally, 10 fractions were collected and dried under a vacuum.

#### 2.2.5 LC–MS/MS analysis

For transition library construction, shotgun proteomics analyses were performed using an EASY‐nLCTM 1200 UHPLC system (Thermo Fisher) coupled with an Q Exactive HF (X) mass spectrometer (Thermo Fisher) operating in the data‐dependent acquisition (DDA) mode. The sample was injected into a home‐made C18 Nano‐Trap column (2 cm × 75 μm, 3 μm). Mobile phase A (100% water, 0.1% formic acid) and B (80% acetonitrile, 0.1% formic acid) were prepared. Peptides were separated in a home‐made analytical column (15 cm × 150 μm, 1.9 μm), using a linear gradient elution. The separated peptides were analysed by Q Exactive HF (X) mass spectrometer (Thermo Fisher) with an ion source of Nanospray Flex™ (ESI, spray voltage of 2.5 kV and ion transport capillary temperature of 320°C. A full scan range from m/z 407 to 1500 was performed with a resolution of 60 000 (at m/z 200). The automatic gain control (AGC) target value was 3 × 10^6^, and a maximum ion injection time was 20 ms. The top 40 precursors of the highest abundant in the full scan were selected and fragmented by higher energy collisional dissociation (HCD) and analysed in MS/MS, where resolution was 15 000 (at m/z 200), the automatic gain control (AGC) target value was 5 × 10^4^, the maximum ion injection time was 45 ms, a normalized collision energy was set as 32%, an intensity threshold was 2.2 × 10^4^ and the dynamic exclusion parameter was 20 s. The raw data of MS detection was named as ‘.raw’.

### 2.3 Data analysis

#### 2.3.1 Identification and quantification of protein

The resulting spectra from each run were searched separately according to the protein database by the search engines: Proteome Discoverer 2.2 (PD 2.2, Thermo). The searched parameters are set as follows: Mass tolerance for precursor ion was 10 ppm, and mass tolerance for product ion was 0.02 Da. Carbamidomethyl was specified as fixed modifications, oxidation of methionine (M) and iTRAQ plex were specified as dynamic modification and acetylation and iTRAQ plex were specified as N‐terminal modification in PD 2.2. A maximum of two miscleavage sites were allowed. In order to improve the quality of analysis results, the software PD 2.2 further filtered the retrieval results: peptide spectrum matches (PSMs) with a credibility of more than 99% were identified PSMs. The identified protein contains at least 1 unique peptide. The identified PSMs and protein were retained and performed with FDR no more than 1.0%. The protein quantitation results were statistically analysed by *T*‐test. The proteins, whose quantitation was significantly different between experimental and control groups (*p* < 0.05, FC > 1.2 or FC < 0.83 [fold change, FC]), were defined as differentially expressed proteins (DEP).

#### 2.3.2 The functional analysis of proteins and DEPs

Gene Ontology (GO) functional analysis was conducted using the InterProScan program against the non‐redundant protein database (including Pfam, PRINTS, ProDom, SMART, ProSite, PANTHER), and the databases of KEGG (Kyoto Encyclopedia of Genes and Genomes) were used to analyse the protein family and pathway. DPEs were used for Volcanic map analysis, cluster heat map analysis and enrichment analysis of GO, KEGG. The probable protein–protein interactions were predicted using the STRING‐db server (http://string.embl.de/).

### 2.4 Validation of iTRAQ data for selected proteins by parallel reaction monitoring (PRM) assay

PRM is a new development of targeted mass spectrometry, which has higher specificity and sensitivity than selected reaction monitoring and has been widely used in the quantification and detection of target proteins. In this study, the protein expression profile obtained by iTRAQ‐based proteomics analysis was confirmed by PRM‐MS analysis to quantify the expression levels of some selected proteins using additional samples. Two candidate proteins related to drug addiction were selected for PRM analysis. Targeted MS analysis using PRM was performed on a TripleTOF EASY‐nLC™ 1200 nano‐UHPLC system. Then, mobile phase A (100% water, 0.1% formic acid) and B (80% acetonitrile, 0.1% formic acid) were prepared. The same amount of trypsin treated‐peptide of each sample was taken and spiked with an equal amount of the labelled peptide DSPSAPVN**V**TVR (red bold V for heavy isotope labelling) as an internal standard. The UHPLC system was upgraded with Easy‐NLCTM 1200 nM, with a pre‐column of Acclaim Pepmap 100 C18 Nano‐Trap (2 cm × 75 μm, 3 μm) and a self‐made analysis column (15 cm × 150 μm, 1.9 μm). Q Exactive™ HF‐X mass spectrometer and NanoPray Flex™ (ESI) ion source were used. The ion spray voltage was set at 2.4 kV, and the temperature of the ion transport tube was set at 320°C. A full scan with a PRM scan was used for mass spectrometry. The resolution of full‐scan mass spectrometry was set as 60 000 (200 m/z), the maximum capacity of C‐TRAP was 3 × 10^6^ and the maximum injection time of C‐TRAP was 20 ms. The PRM resolution was set as 30 000 (200 m/z), the maximum C‐TRAP capacity was 5 × 10^4^, the maximum C‐TRAP injection time was set as 80 ms, the peptide fragmentation collision energy was set as 27% and the raw data of mass spectrometry detection was generated (.RAW). The offline data was analysed by Skyline software, and the peak area was corrected using the internal standard peptide.

## 3 RESULTS

### 3.1 Proteins identified based on iTRAQ

**FIGURE 1:**
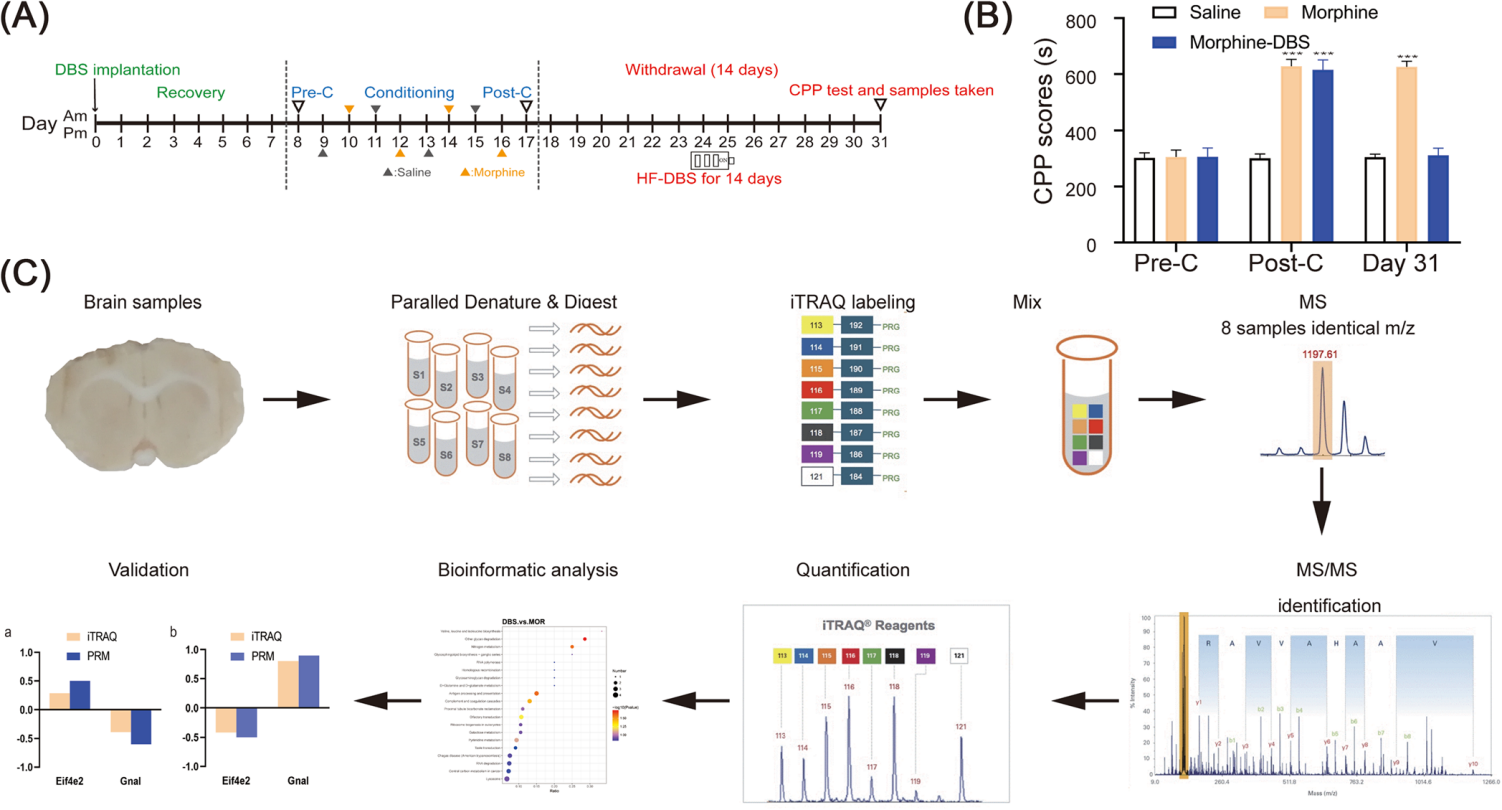
(A) Experimental timeline from DBS implantation until relapse test and samples taken. On day 0, the rats in the morphine–DBS group received DBS implantation and recovered for 7 days. From day 8 to day 17, all animals from the three groups underwent CPP training (including pre‐C, conditioning and post‐C) and proceeded into the withdrawal phase from day 18 and day 31 during which rats abstained from the drug. Chronic HF‐electrical stimulation was applied in the DBS‐implanted rats for 14 days during the withdrawal stage. On day 31 post‐withdrawal, all experimental animals conducted CPP tests and were immediately decapitated for sample collection. (B) Eight‐day alternative morphine injection induced significant morphine side preference in morphine and morphine–DBS rats on post‐C day. Saline did not affect the place preference in the saline group. Chronic high‐frequency DBS of the anterior insula blocked the relapse of morphine‐conditioned place preference in morphine–DBS animals on day 31 during the withdrawal stage. The CPP scores were comparable on day 17 and day 31 in the morphine groups. A two‐way repeated measures analysis of variance (RM‐ANOVA) followed by the Bonferroni post hoc test was applied. ***p < 0.001 versus saline group. *N* = 6 for all groups. (C) Experimental workflow diagram for preparation and iTRAQ analysis of insula samples. The insula samples were collected, and total proteins were extracted. Equal amounts of proteins from the samples were digested and labelled with eight‐plex iTRAQ reagents (113–119, 121). The labelled peptides were mixed in equal amounts and analysed using 2D LC–MS/MS analysis. The search engine (Proteome Discoverer 2.2) was used to identify and quantify the proteins. Bioinformatic analysis (GO, KEGG pathway) was carried out to examine the differential expression proteins. The differentially expressed proteins of interest were selected and further verified with PRM.

**FIGURE 2:**
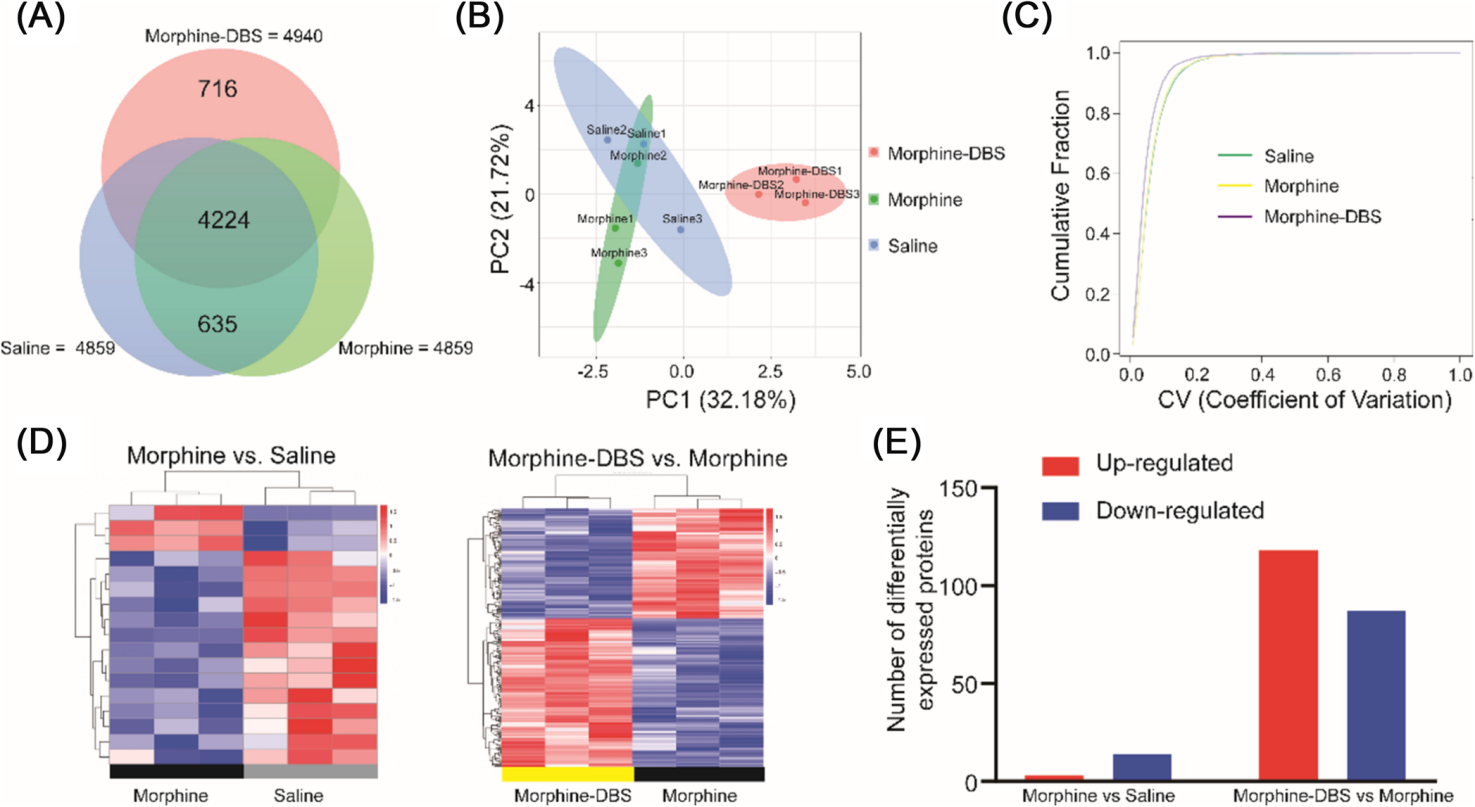
Identification of total proteins and global protein expression patterns in saline, morphine and morphine–DBS groups. (A) Venn diagram showing the proteins detected in three groups. 4224 proteins were commonly identified in three groups and the same proteins were detected in saline and morphine groups, with 716 proteins being identified only in morphine–DBS. (B) Principal component analysis (PCA) of protein expression in saline, morphine and morphine–DBS groups. There was a significant difference in total protein expression between saline or morphine and DBS groups. (C) Repeatability analysis across three groups using coefficient of variance (CV). The horizontal axis represents the CV value, longitudinal axis represents the cumulative CV values of all proteins in the corresponding samples. (D) Numbers of differentially expressed proteins in two comparisons. The numbers above the bars show the quantity of up‐regulated (red) and down‐regulated (blue) expression proteins. (E) Heat map comparing anterior insula protein expression in morphine versus saline and DBS versus morphine groups. Rows represent different proteins clustering and columns represent different samples clustering. The expression level of proteins is represented by a colour scale (top right) going from low (blue) to high (red).

The experimental design was illustrated in Figure 1A–C. In order to understand the proteomic changes in morphine addiction withdrawal and DBS treatment, we used the quantitative proteomic approach based on iTRAQ coupled with 2D‐LC MS/MS (Figure 1C). A total of 4650 non‐redundant proteins were identified by global proteomic analysis with >99% confidence in correct sequence identification. There were 4224 proteins commonly identified among saline, morphine and morphine–DBS groups. There were 716 proteins detected only in morphine–DBS group (Figure 2A). PCA can reveal the underlying structure of the data in a way that best explains the variance in the original data set. In the present study, the samples are clustered respectively and separated from each other in the PCA score plot, which suggested that morphine or HF‐DBS intervention for the variations in the proteins identified (Figure 2B). The quality of the proteomic dataset was evaluated by the coefficient of variance (CV) analysis; the results suggested that there were no biases toward samples of three groups, demonstrating good reproducibility of biological replicates (Figure 2C). Protein with a significant quantitative difference (*p* < 0.05, FC ≥ 1.2 or FC ≤ 0.83) was defined as differential expression protein (DEP). We also identified the significance and magnitude of change in the expression of DEPs across two comparisons, using a heatmap of a hierarchical clustering analysis (HCA) and showing a unique and diverse change pattern of protein abundance in each group (Figure 2D). Our results showed that there were 14 down‐regulated and three up‐regulated proteins in the morphine versus saline group, as well as 118 up‐regulated and 87 down‐regulated proteins in DBS versus morphine group respectively (Figure 2E).

### 3.2 Bioinformatics analysis

**FIGURE 3:**
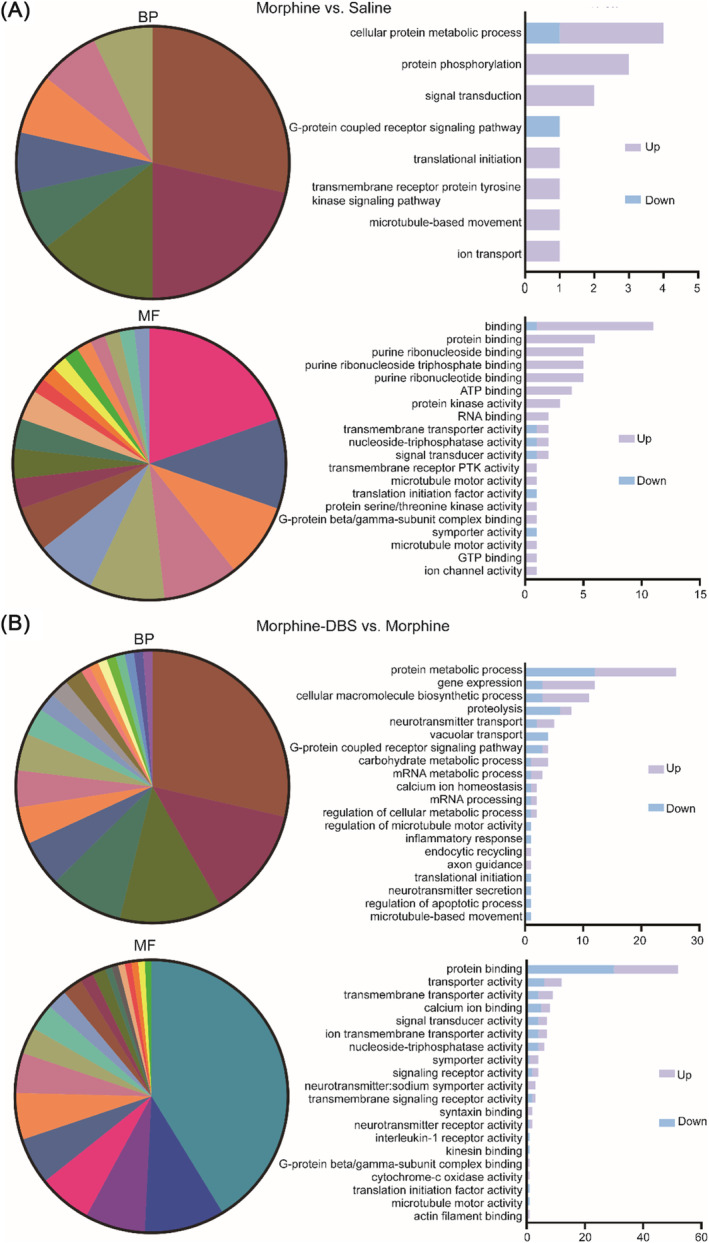
Gene ontology (GO) annotation and functional classification of differential expression proteins. (A) The enriched GO terms of DEPs under biological process (BP) and molecular function (MF) in morphine versus saline. (B) Differential proteins in the morphine–DBS group compared to the morphine group were analysed through GO in BP and MF.

In order to fully understand the above DEPs, GO analysis is carried out using InterProScan software. A total of 41 GO terms were obtained from differential proteins between morphine and saline groups, including eight biological process terms, three cellular component terms and 30 molecular function terms. The DEPs between DBS and morphine groups resulted in 221 GO terms, including 97 biological process terms, 24 cellular component terms and 100 molecular function terms. The common enriched GO terms from two sets of differential proteins under biological process included translational initiation, G‐protein coupled receptor signalling pathway, microtubule‐based movement (Figure 3A and B). The common enriched GO terms under molecular function included symporter activity, carbonate dehydratase activity, signal transducer activity, G‐protein beta/gamma‐subunit complex binding, protein binding, translation initiation factor activity, microtubule motor activity, transmembrane transporter activity, hydrolase activity, protein binding and nucleoside–triphosphatase activity (Figure 3A and B).

**FIGURE 4:**
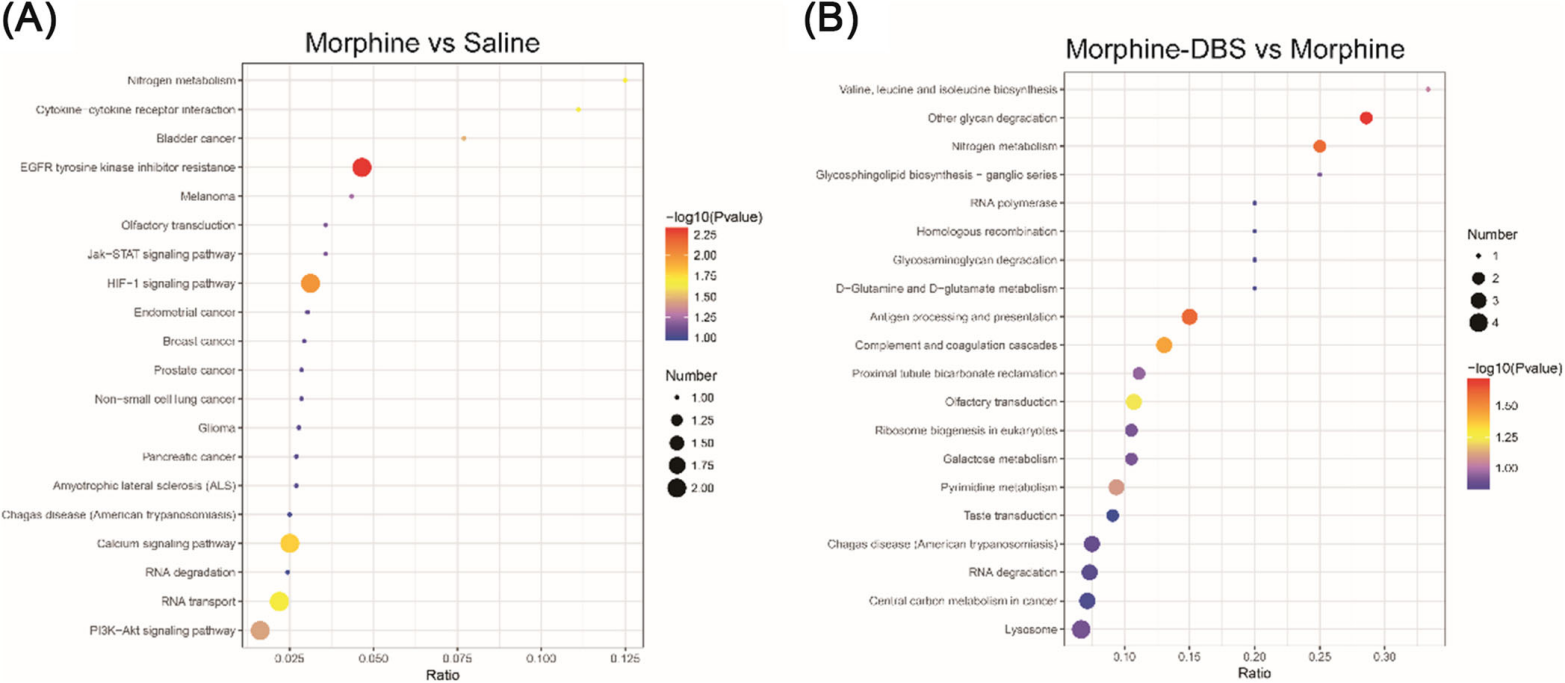
Twenty enriched KEGG pathways in morphine versus saline (A) and morphine–DBS versus morphine (B).

DEPs were then mapped to the reference pathway in the KEGG database to determine the biological path involved in morphine addiction withdrawal and DBS therapy. Among these 17 identified DEPs from morphine versus saline, only seven DEPs (five down‐regulated and two up‐regulated) had a KEGG Orthology (KO) ID and were involved in 52 pathways. Among the 205 DEPs identified from DBS versus morphine, 94 DEPs (70 down‐regulated and 24 up‐regulated) were mapped to 106 pathways. Many enriched pathways are close to addiction including PI3K‐Akt signalling pathway, calcium signalling pathway, mTOR signalling pathway and nicotine addiction (Figure 4).

### 3.3 Protein–protein interaction analysis of DEPs

**FIGURE 5:**
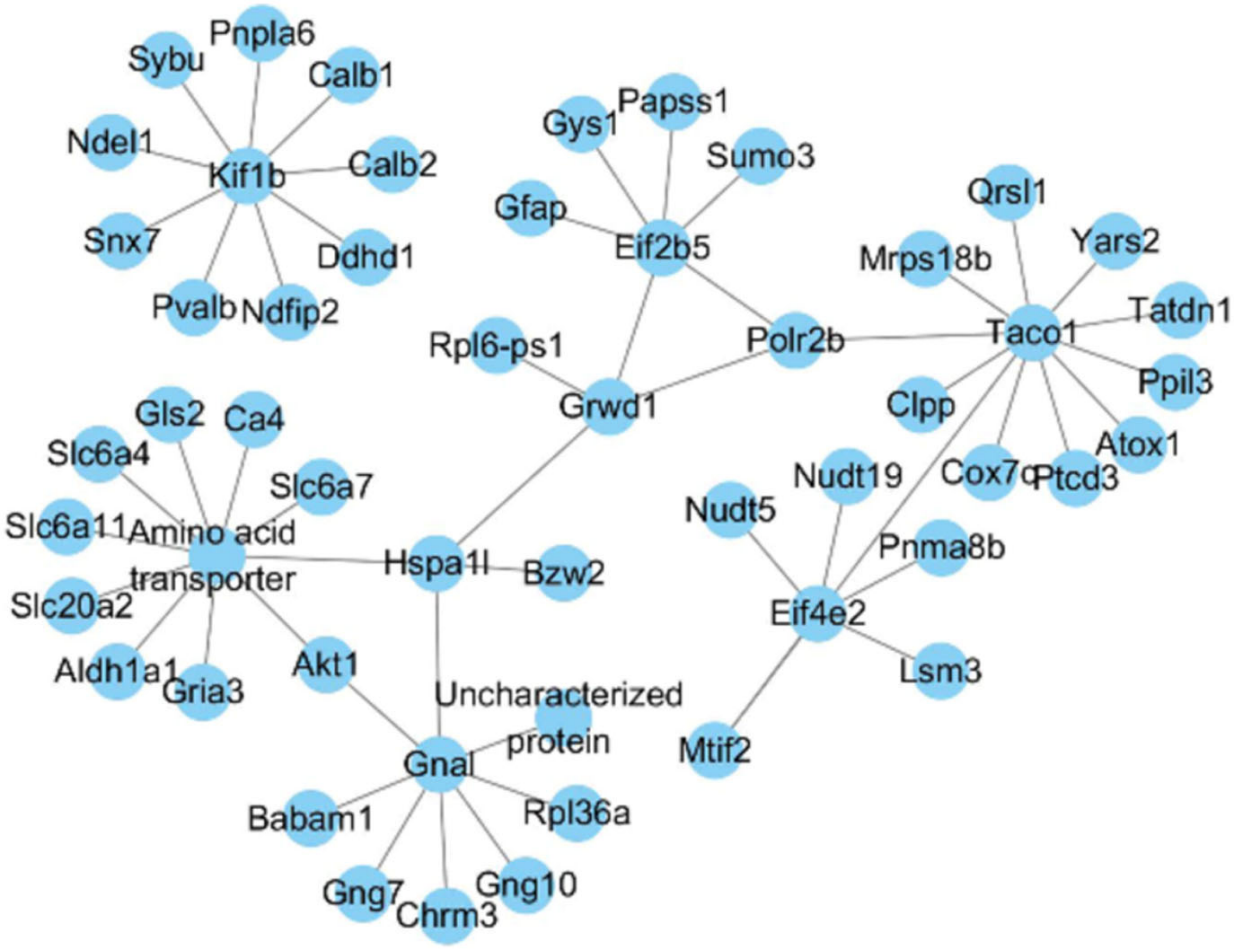
Protein–protein interactions analysis of eight common DEPs in morphine versus saline and morphine–DBS versus morphine and their directly interacted DEPs.

The protein–protein interaction (PPI) network of the DEPs identified in present study based on STRING action scores was analysed. The PPI of eight common DEPs is shown in Figure 5, revealing that guanine nucleotide‐binding protein G (olf) subunit alpha (Gnal), eukaryotic translation initiation factor 4E family member 2 (Eif4e2), kinesin‐like protein KIF1B (kif1b), amino acid transporter and translational activator of cytochrome c oxidase 1 (taco1) were present at hub positions of the networks (Figure 5).

### 3.4 PRM verification of selected DEPs

**FIGURE 6:**
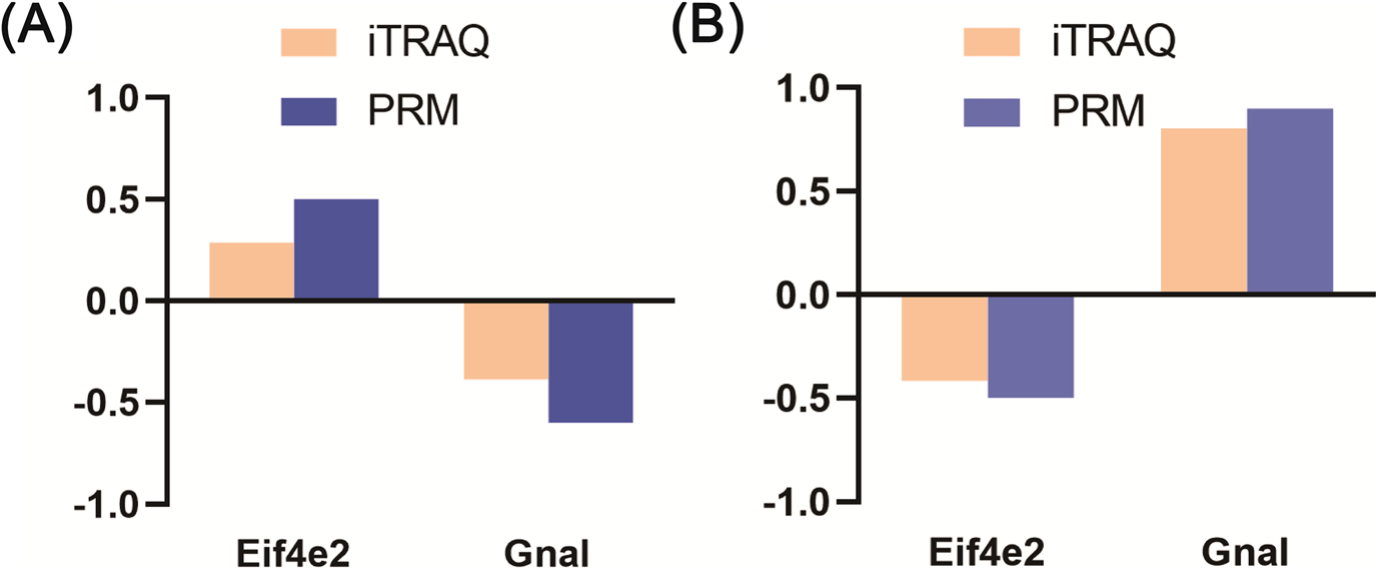
PRM verification of proteins identified by iTRAQ analysis. Two proteins were selected for validation of the iTRAQ data. Data of iTRAQ were verified by PRM in morphine versus saline (A) and morphine–DBS versus morphine (B).

Two candidate proteins (eukaryotic translation initiation factor 4E family member 2, guanine nucleotide‐binding protein G (olf) subunit alpha) were of particular interest and selected because they were mapped to several pathways involved in addiction. As shown in Figure 6, the expression of proteins verified by PRM was in accordance with proteomic data.

## 4 DISCUSSION

In the current study, we utilized iTRAQ‐based proteomic analysis and determined the differential regulatory proteins in the insula during the morphine addiction withdrawal and DBS treatment in morphine addictive animal model. HF‐DBS has been shown to regulate neuronal activity in the stimulated area, alter the expression of neural activity genes and affect the occurrence and development of addiction, as confirmed in our previous work. Among them, there were 17 DEPs between morphine and saline groups and 205 when morphine–DBS compared to the morphine group (Data S1). GO and KEGG analysis showed that DEPs were related to multiple signalling pathways including PI3K‐Akt pathway. Two DEPs involved in DBS therapy are of particular interest: Eif4e2 and Gnal, due to their important role in signalling pathways associated with addiction from relevant literature. PPI network analysis also revealed that Eif4e2 and Gnal were shown at the hub positions, which interacted with other important proteins.

Eukaryotic translation initiation factor 4E (Eif4e) serves as a central player in the complex processes of eukaryotic translation initiation and regulation. Eif4e2 is recognized as one of its family members.^32^ Eif4e's specialized role involves binding to the 7‐methylguanosine triphosphate(m7GpppG) cap structure found at the 5′ end of mRNA. This binding profoundly affects mRNA metabolism, processing, transport and translation, making it a crucial contributor to the initial stage of protein synthesis.^33^, ^34^, ^35^ Recent research has shown that Eif4e‐dependent translation is important in nerve function, particularly in the context of neurodevelopment and neuropsychiatric diseases.^36^, ^37^, ^38^ Notably, Eif4e's activity is strictly regulated by various factors, such as hormones, growth factors, cytokines and extracellular stimuli. It is mainly involved in two key signalling pathways: MAPK/ERK and PI3K/mTOR.^39^, ^40^

Numerous studies have consistently demonstrated that u‐opioid receptor induces activation of PI3K/Akt/mTOR pathway.^41^, ^42^, ^43^, ^44^ In the context of morphine‐induced CPP, the PI3K/Akt–mTOR signalling pathway activated by a μ‐opioid receptor in the CA3 region of the hippocampus plays an important role in the acquisition of CPP.^42^, ^43^ We demonstrate that morphine‐regulated DEPs in the insular cortex are enriched in PI3K‐Akt pathway that is consistent with previous studies. These findings collectively highlight the pivotal role of the PI3K/Akt–mTOR pathway in the development of drug addiction. Furthermore, interventions targeting this pathway offer a promising and novel approach to address issues related to drug abuse.^45^

Guanine nucleotide‐binding protein G (olf) subunit α (Gαolf), encoded by GNAL gene located on chromosome 18p11, belongs to the family of GTP‐binding proteins (G protein). These proteins serve as intermediaries, connecting G protein‐coupled receptors (GPCRs) to adenylate cyclase.^46^, ^47^ The heterotrimeric G protein complex comprises three subunits: α, β and γ.^48^ In humans, there are 21 G21 Gα, 6Gβ and 12 Gγ units identified. G proteins can be categorized into four subfamilies (Gαs, Gαi/o, Gαq and Gα12) based on the sequence similarity of Gα subunits.^49^ Gα (olf), with an 88% homology to Gαs, is considered to be a member of the Gαs family.^50^ G‐proteins play an important role in transmitting external signals to the interior of cells, when activated by GPCRs localized on the cell membrane.^51^ They are involved in a wide array of physiological functions and are implicated in numerous pathological conditions. In our study, Gαolf was mapped to the dopaminergic synapse pathway (map 04728) using the Kyoto Encyclopedia of Genes and Genomes (KEGG) reference database (http://www.genome.jp/kegg/pathway.html). The dopaminergic pathways are associated with processes such as reward sensitivity, incentive motivation, conditioning, and control.^52^ Dysregulation of dopamine activity can lead to loss of control over intake and continued consumption despite adverse consequences, which is commonly observed in addiction and obesity.^53^ Gαolf functions as a modulator during neurotransmission and is closely associated with dopamine signalling.^46^ Dopamine primarily exerts its effects through D1‐like (D1, D5) and D2‐like (D2, D3, D4) receptors, which, in turn, regulate the activation or inhibition of cAMP accumulation, via Gs/olf or Gi/o proteins, respectively.^54^ Furthermore, Gαolf was also mapped to the calcium signalling pathway in our study. GPCRs and G‐protein effectors are intricately involved in calcium signalling. Various physiological stimuli, including odours, light, metal ions, peptide hormones and neurotransmitters, induce a conformational change in GPCRs, activating heterotrimeric G‐proteins and downstream effectors like adenylate cyclase and phospholipase C. These effectors generate diffusible second messengers that trigger intracellular changes including alterations in cytosolic calcium concentration.^55^

Calcium signalling is broadly implicated in regulating various aspects of cell function^56^ and can be influenced by tissue injury, resulting in alterations in cell functions.^57^ Dysregulation of calcium signalling and subsequent changes BDNF expression may be linked to drug addiction.^58^ In a proteomic analysis using iTRAQ, DEPs associated with calcium‐mediated signalling were identified in nicotine‐induced CPP rats.^59^ Notably, a pathway analysis conducted in a genome‐wide association study (GWAS) investigating opioid dependence in African‐American and European‐American populations highlighted calcium signalling as the most significant pathways,^60^ offering potential insights into novel therapeutic and preventive strategies for drug addiction.

A gene network analysis employing the bioinformatics tool IPA, to identify commonly shared genes associated with alcohol, smoking and opioid addiction, revealed several top canonical pathways, including calcium signalling. GPCR signalling, cAMP‐mediated signalling, GABA receptor signalling and Gαi signalling. These pathways have been consistently linked to substance addiction in existing literature.^61^ Together with prior research, our study identifies Gαolf, an effector of GPCR, as a key player in morphine addiction mediated through the dopaminergic synapse pathway and calcium signalling pathways. Furthermore, our study demonstrates that DBS reverses the expression of Gαolf in morphine‐addicted animals, shedding light on the potential mechanism of DBS in treating drug addiction.

In summary, our iTRAQ‐based proteomic analysis revealed 17 differential expressed proteins in the insula of morphine‐addicted rats following withdrawal. DBS treatment for drug addiction has been extensively analysed in our team's previous published article. The insula plays a crucial role in drug addiction, and compared to other brain regions or nuclei, the insula is larger, making it easier for high‐frequency DBS to receive clear stimulation interventions. Of note, eight of these proteins exhibited changes with DBS intervention, involving signal transduction, translation regulation and neurotransmitter transmission. This contributes to a deeper understanding of morphine addiction pathogenesis and the molecular mechanisms underlying DBS therapy. Additionally, the proteins identified in this study may serve as valuable therapeutic targets, contributing to the advancement of DBS‐based treatments for drug addiction.

## AUTHOR CONTRIBUTIONS

Haigang Chang performed the experiments, analysed the data and drafted the manuscript. Yaxiao Wang and Lei Hui conducted the study, including data collection and analysis. Yuling Diao and Pengju Ma contributed to the manuscript drafting. Feng Wang designed the experiments and contributed to manuscript drafting. Xiangsheng Li supervised the study. All authors critically reviewed the content and approved the final version for publication.

## CONFLICT OF INTEREST STATEMENT

The authors declare that there are no financial and personal relationships with other people or organizations that can inappropriately influence our work, and there is no professional or other personal interest of any nature or kind in any product, service and/or company that could be construed as influencing the position presented in, or the review of, the manuscript entitled.

## Supporting information


**Data S1.** Supporting Information.

## Data Availability

The data that support the findings of this study are available from the corresponding author upon reasonable request.
