# Supplementary material for: iTRAQ proteomic analysis of the anterior insula in morphine‐induced conditioned place preference rats with high‐frequency deep brain stimulation intervention
Source: Addict Biol. 2025 Jan 21;30(1):e70014. doi: 10.1111/adb.70014 (PMC11747870; doi:10.1111/adb.70014)
Supplement: Supplementary file 1 — Data S1. Supporting Information. [file ADB-30-e70014-s001.doc]

**The functions of the 17 proteins**

| **Protein ID** | **Protein names** | **Gene names** | **Function** |
| --- | --- | --- | --- |
| A0A0G2K1R5 | CaM kinase-like vesicle-associated protein | *Camkv* | Does not appear to have detectable kinase activity. |
| A0A0G2K526 | Guanine nucleotide-binding protein G(olf) subunit alpha | *Gnal* | Guanine nucleotide-binding proteins (G proteins) are involved as modulators or transducers in various transmembrane signaling systems. G(olf) alpha mediates signal transduction within the olfactory neuroepithelium and the basal ganglia. May be involved in some aspect of visual transduction, and in mediating the effect of one or more hormones/neurotransmitters. |
| A0A0G2K933 | Eukaryotic translation initiation factor 4E family member 2 | *Eif4e2* | Recognizes and binds the 7-methylguanosine-containing mRNA cap during an early step in the initiation. Acts as a repressor of translation initiation 4. In contrast to EIF4E, it is unable to bind eIF4G (EIF4G1, EIF4G2 or EIF4G3), suggesting that it acts by competing with EIF4E and block assembly of eIF4F at the cap (By similarity). In P-bodies, component of a complex that promotes miRNA-mediated translational repression |
| A0A140TAH3 | Glutamate-rich WD repeat-containing protein 1 | *Grwd1* | Histone binding-protein that regulates chromatin dynamics and minichromosome maintenance (MCM) loading at replication origins, possibly by promoting chromatin openness |
| B2RYT9 | Translational activator of cytochrome c oxidase 1 | *Taco1* | Acts as a translational activator of mitochondrially-encoded cytochrome c oxidase 1. |
| D3ZC46 | Transcription factor 25 | *Tcf25* | May play a role in cell death control. Acts as a transcriptional repressor. Has been shown to repress transcription of SRF in vitro and so may play a role in heart development |
| E7CXR8 | Receptor protein-tyrosine kinase | *Egfr* | Receptor tyrosine kinase binding ligands of the EGF family and activating several signaling cascades to convert extracellular cues into appropriate cellular responses |
| M0RBQ1 | mRNA-decapping enzyme 2-like | *Dcp2* | Decapping metalloenzyme that catalyzes the cleavage of the cap structure on mRNAs. Removes the 7-methyl guanine cap structure from mRNA molecules, yielding a 5'-phosphorylated mRNA fragment and 7m-GDP |
| M0RCY7 | UFM1-specific peptidase 1 | *Ufsp1* | This gene encodes a protein that is similar to other Ufm1-specific proteases. Studies in mouse determined that Ufsp1 releases Ufm1 (ubiquitin-fold modifier 1) from its bound conjugated complexes which also makes it into an active form. Because the human UFSP1 protein is shorter on the N-terminus and lacks a conserved Cys active site, it is predicted to be non-functional |
| O88658 | Kinesin-like protein KIF1B | *Kif1b* | Motor for anterograde transport of mitochondria. Has a microtubule plus end-directed motility. Isoform 2 is required for induction of neuronal apoptosis. |
| P05545 | Serine protease inhibitor A3K | *Serpina3k* | Binds to and inhibits kallikreins. Inhibits trypsin but not chymotrypsin or elastase. |
| P14141 | Carbonic anhydrase 3 | *Ca3* | Reversible hydration of carbon dioxide. |
| Q63638 | Striated muscle-specific serine/threonine-protein kinase | *Speg* | Isoform 3 may have a role in regulating the growth and differentiation of arterial smooth muscle cells. |
| Q64350 | Translation initiation factor eIF-2B subunit epsilon | *Eif2b5* | Catalyzes the exchange of eukaryotic initiation factor 2-bound GDP for GTP. |
| Q91XV6 | FXYD domain-containing ion transport regulator 6 | *Fxyd6* | May be involved in forming the receptor site for cardiac glycoside binding or may modulate the transport function of the sodium ATPase. |
| Q9WTT7 | Basic leucine zipper and W2 domain-containing protein 2 | *Bzw2* | Translation initiation regulator which represses non-AUG initiated translation and repeat-associated non-AUG (RAN) initiated translation by acting as a competitive inhibitor of eukaryotic translation initiation factor 5 (EIF5) function |
| Q8R462 | Amino acid transporter (Fragment) |  | acidic amino acid transmembrane transporter activity |

修改后

| **Protein ID** | **Protein names** | **Gene names** | **Function** |
| --- | --- | --- | --- |
| A0A0G2K1R5 | CaM kinase-like vesicle-associated protein | *Camkv* | There appears to be no detectable kinase activity. |
| A0A0G2K526 | Guanine nucleotide-binding protein G(olf) subunit alpha | *Gnal* | Guanine nucleotide binding proteins (G proteins) function as regulators or converter in various transmembrane signaling systems. G (olf) α mediates signal transduction within the olfactory neuroepithelium and basal ganglia. May be involved in some aspects of visual transduction and mediate the action of one or more hormones / neurotransmitters. |
| A0A0G2K933 | Eukaryotic translation initiation factor 4E family member 2 | *Eif4e2* | Identify and bind mRNA caps containing 7-methylguanosine during early steps of initiation. Act as a repressor of translation initiation 4. In contrast to EIF 4 E, it cannot bind eIF 4 G (EIF4G1, EIF4G2, or EIF4G3), suggesting that it acts by competing with EIF4E and blocking the assembly of eIF4F at the cap. In the P-body, components of the complex that promote miRNA-mediated translational repression |
| A0A140TAH3 | Glutamate-rich WD repeat-containing protein 1 | *Grwd1* | Histone-binding proteins, which may regulate chromatin dynamics at replication origins and microchromosome maintenance (MCM) loading by promoting chromatin openness |
| B2RYT9 | Translational activator of cytochrome c oxidase 1 | *Taco1* | As a translation activator of cytochrome c oxidase 1 encoded by mitochondria. |
| D3ZC46 | Transcription factor 25 | *Tcf25* | May play a role in cell death control. |
| E7CXR8 | Receptor protein-tyrosine kinase | *Egfr* | Receptor tyrosine kinases bind the ligands of the EGF family and activate several signaling cascades to convert extracellular cues into appropriate cellular responses |
| M0RBQ1 | mRNA-decapping enzyme 2-like | *Dcp2* | Uncoating metalloenzyme that catalyzes the cleavage of the cap structure on a messenger ribonucleic acid.  Removal of the 7-methyl guanine cap structure from the messenger ribonucleotide molecule produces 5 ′ -phosphorylated messenger ribonucleotide fragments and 7 million GDP |
| M0RCY7 | UFM1-specific peptidase 1 | *Ufsp1* | This gene encodes a protein that is similar to the other Ufm 1-specific proteases. The human UFSP 1 protein is predicted to be nonfunctional due to its short N terminus and its lack of the conserved Cys active site |
| O88658 | Kinesin-like protein KIF1B | *Kif1b* | It is a motor for anterograde transport of the mitochondria, with microtubules and end-directed movements.  Its induction of neuronal apoptosis requires isoforms 2. |
| P05545 | Serine protease inhibitor A3K | *Serpina3k* | Bind and inhibit kallikreins releasase and inhibit trypsin |
| P14141 | Carbonic anhydrase 3 | *Ca3* | Reversible hydration of carbon dioxide. |
| Q63638 | Striated muscle-specific serine/threonine-protein kinase | *Speg* | Isoform 3 may play a role in regulating the growth and differentiation of arterial smooth muscle cells. |
| Q64350 | Translation initiation factor eIF-2B subunit epsilon | *Eif2b5* | Catalyzes the exchange of eukaryotic initiation factor 2-bound GDP for GTP. |
| Q91XV6 | FXYD domain-containing ion transport regulator 6 | *Fxyd6* | Could be involved in forming the receptor site for cardiac glycoside binding, or may regulate the transport function of the sodium ATP enzyme. |
| Q9WTT7 | Basic leucine zipper and W2 domain-containing protein 2 | *Bzw2* | Translation initiation regulators, inhibiting non-AUG initiated translation and repeat-related non-AUG (RAN) initiated translation by acting as competitive inhibitors of eukaryotic translation initiation factor 5 (EIF5) function |
| Q8R462 | Amino acid transporter (Fragment) |  | Activity of acidic amino acid transmembrane transport proteins |

修改前后

| **Function** | **Function** |
| --- | --- |
| Does not appear to have detectable kinase activity. | There appears to be no detectable kinase activity. |
| Guanine nucleotide-binding proteins (G proteins) are involved as modulators or transducers in various transmembrane signaling systems. G(olf) alpha mediates signal transduction within the olfactory neuroepithelium and the basal ganglia. May be involved in some aspect of visual transduction, and in mediating the effect of one or more hormones/neurotransmitters. | Guanine nucleotide binding proteins (G proteins) function as regulators or converter in various transmembrane signaling systems. G (olf) α mediates signal transduction within the olfactory neuroepithelium and basal ganglia. May be involved in some aspects of visual transduction and mediate the action of one or more hormones / neurotransmitters. |
| Recognizes and binds the 7-methylguanosine-containing mRNA cap during an early step in the initiation. Acts as a repressor of translation initiation 4. In contrast to EIF4E, it is unable to bind eIF4G (EIF4G1, EIF4G2 or EIF4G3), suggesting that it acts by competing with EIF4E and block assembly of eIF4F at the cap (By similarity). In P-bodies, component of a complex that promotes miRNA-mediated translational repression | Identify and bind mRNA caps containing 7-methylguanosine during early steps of initiation. Act as a repressor of translation initiation 4. In contrast to EIF 4 E, it cannot bind eIF 4 G (EIF4G1, EIF4G2, or EIF4G3), suggesting that it acts by competing with EIF4E and blocking the assembly of eIF4F at the cap. In the P-body, components of the complex that promote miRNA-mediated translational repression |
| Histone binding-protein that regulates chromatin dynamics and minichromosome maintenance (MCM) loading at replication origins, possibly by promoting chromatin openness | Histone-binding proteins, which may regulate chromatin dynamics at replication origins and microchromosome maintenance (MCM) loading by promoting chromatin openness |
| Acts as a translational activator of mitochondrially-encoded cytochrome c oxidase 1. | As a translation activator of cytochrome c oxidase 1 encoded by mitochondria. |
| May play a role in cell death control. Acts as a transcriptional repressor. Has been shown to repress transcription of SRF in vitro and so may play a role in heart development | May play a role in cell death control. |
| Receptor tyrosine kinase binding ligands of the EGF family and activating several signaling cascades to convert extracellular cues into appropriate cellular responses | Receptor tyrosine kinases bind the ligands of the EGF family and activate several signaling cascades to convert extracellular cues into appropriate cellular responses |
| Decapping metalloenzyme that catalyzes the cleavage of the cap structure on mRNAs. Removes the 7-methyl guanine cap structure from mRNA molecules, yielding a 5'-phosphorylated mRNA fragment and 7m-GDP | Uncoating metalloenzyme that catalyzes the cleavage of the cap structure on a messenger ribonucleic acid.  Removal of the 7-methyl guanine cap structure from the messenger ribonucleotide molecule produces 5 ′ -phosphorylated messenger ribonucleotide fragments and 7 million GDP |
| This gene encodes a protein that is similar to other Ufm1-specific proteases. Studies in mouse determined that Ufsp1 releases Ufm1 (ubiquitin-fold modifier 1) from its bound conjugated complexes which also makes it into an active form. Because the human UFSP1 protein is shorter on the N-terminus and lacks a conserved Cys active site, it is predicted to be non-functional | This gene encodes a protein that is similar to the other Ufm 1-specific proteases. The human UFSP 1 protein is predicted to be nonfunctional due to its short N terminus and its lack of the conserved Cys active site |
| Motor for anterograde transport of mitochondria. Has a microtubule plus end-directed motility. Isoform 2 is required for induction of neuronal apoptosis. | It is a motor for anterograde transport of the mitochondria, with microtubules and end-directed movements.  Its induction of neuronal apoptosis requires isoforms 2. |
| Binds to and inhibits kallikreins. Inhibits trypsin but not chymotrypsin or elastase. | Bind and inhibit kallikreins releasase and inhibit trypsin |
| Reversible hydration of carbon dioxide. | Reversible hydration of carbon dioxide. |
| Isoform 3 may have a role in regulating the growth and differentiation of arterial smooth muscle cells. | Isoform 3 may play a role in regulating the growth and differentiation of arterial smooth muscle cells. |
| Catalyzes the exchange of eukaryotic initiation factor 2-bound GDP for GTP. | Catalyzes the exchange of eukaryotic initiation factor 2-bound GDP for GTP. |
| May be involved in forming the receptor site for cardiac glycoside binding or may modulate the transport function of the sodium ATPase. | Could be involved in forming the receptor site for cardiac glycoside binding, or may regulate the transport function of the sodium ATP enzyme. |
| Translation initiation regulator which represses non-AUG initiated translation and repeat-associated non-AUG (RAN) initiated translation by acting as a competitive inhibitor of eukaryotic translation initiation factor 5 (EIF5) function | Translation initiation regulators, inhibiting non-AUG initiated translation and repeat-related non-AUG (RAN) initiated translation by acting as competitive inhibitors of eukaryotic translation initiation factor 5 (EIF5) function |
| acidic amino acid transmembrane transporter activity | Activity of acidic amino acid transmembrane transport proteins |
